# Telomerase Activity in Melanoma: Impact on Cancer Cell Proliferation Kinetics, Tumor Progression, and Clinical Therapeutic Strategies—A Scoping Review

**DOI:** 10.3390/curroncol33020074

**Published:** 2026-01-27

**Authors:** Omar Alqaisi, Guy Storme, Amaechi Dennis, Mohammed Dibas, Lorent Sijarina, Liburn Grabovci, Shima Al-Zghoul, Edward Yu, Patricia Tai

**Affiliations:** 1 Nursing Department, Al-Zaytoonah University, Airport Street, Amman 11733, Jordan; 2 Department Radiation Oncology, Universities Ziekenhuis Brussel, Laarbeeklaan 101, 1090 Jette, Belgium; guy.storme@telenet.be; 3 Department of Biochemistry, Veritas University, Abuja 901101, Nigeria; amaechitoexcel@yahoo.com; 4 Department of Medicine, Faculty of Medicine and Health Sciences, An-Najah National University, Nablus 00970, Palestine; mohd.dibas@gmail.com; 5 Faculty of Medicine, University of Prishtina, 10000 Prishtina, Kosovo; lorentsijarina87@gmail.com (L.S.); liburn.grabovci2@gmail.com (L.G.); 6 Pharmacy Department, Hashemite University, Anjara 13133, Jordan; shima.hassan5520@gmail.com; 7 Department of Oncology, Western University, London, ON N6A 3K7, Canada; eyu@uwo.ca; 8 Department of Oncology, University of Saskatchewan, 105 Administration Place, Saskatoon, SK S7N 5A2, Canada; pat961@usask.ca

**Keywords:** cell proliferation, doubling time, telomerase, scoping review, survival, prognosis, outcome, resistance, target, melanoma

## Abstract

**Background**: Melanoma outcomes have improved in recent years as a result of modern systemic therapies. A major molecular feature of melanoma is abnormal telomerase activation; this is most often caused by telomerase reverse transcriptase (TERT) promoter mutations, which occur in 50–82% of cases and are the most common noncoding alteration in this cancer. Telomerase maintains telomere length, allowing melanoma cells to avoid senescence and continue dividing. However, how telomerase activity influences melanoma cell doubling time remains unclear, and the pathways linking TERT expression to faster cell-cycle progression require further study. Although telomerase inhibitors show promise in preclinical models, their clinical use is limited by delayed cytotoxicity and resistance. **Materials and Methods**: A scoping review was conducted using Scopus, ScienceDirect, MEDLINE/PubMed, and CINAHL (Cumulative Index to Nursing and Allied Health Literature). Keywords included “telomerase,” “melanoma,” “cancer,” “cell proliferation,” and “doubling time,” using Preferred Reporting Items for Systematic Reviews and Meta-Analyses (PRISMA) guidelines. **Results**: Telomerase-related biomarkers were found to correlate with disease stage and survival. Suggested therapeutic strategies include enzyme inhibitors, cytotoxic nucleotide incorporation, telomere destabilization, and immunotherapies such as peptide or dendritic cell vaccines, etc. **Conclusions**: Understanding both telomere-dependent and -independent TERT functions is essential for developing effective biomarkers and therapies that overcome resistance and slow melanoma progression.

## Simple Summary

Melanoma treatment has improved greatly in recent years due to the development of many new cancer drugs. Many melanomas—50–82% of cases—carry changes in the telomerase reverse transcriptase (TERT) gene, which activates telomerase, an enzyme that maintains chromosome ends called telomeres. Normally, telomeres shorten as cells age; however, in melanoma, telomerase stays active, allowing cancer cells to keep dividing. Although telomerase clearly supports tumor growth, its role in how quickly melanoma cells multiply is not fully understood. This review summarizes current research showing that telomerase-related markers can predict disease severity. Scientists are developing telomerase-targeted drugs and immunotherapies, though resistance remains a challenge.

## 1. Introduction

Melanoma remains a significant global health challenge, with incidence rates steadily rising worldwide [1]. Recent epidemiology data indicate that melanoma accounts for approximately 1.5% of all newly diagnosed cancer cases, with age-adjusted incidence rates increasing from 15.1 per 100,000 people in 1999 to 23.0 per 100,000 people in 2021 [1]. As of 2025, melanoma represents one of the most prevalent cancers, with over 816,580 individuals living with a melanoma diagnosis in the United States alone [2]. A defining molecular characteristic of melanoma is the aberrant activation of telomerase, with 69–82% of cutaneous melanoma cases exhibiting detectable telomerase activity [3,4]. This activation is predominantly mediated by telomerase reverse transcriptase (TERT) promoter mutations [5,6], which occur in 50% to 82% in melanoma cases, representing the most common noncoding mutation in this malignancy [7,8].

Telomerase activation represents a critical mechanism enabling replicative immortality in melanoma cells, directly facilitating sustained cancer cell proliferation and tumor progression [9,10]. The enzyme complex maintains telomere length at chromosomal ends, thereby bypassing cellular senescence and apoptosis pathways that normally limit cell division [3,4,11]. The most prevalent TERT promoter mutations (C228T and C250T, located at 124 and 146 pb from the ATG start site) generate de novo binding sites for ETS (Erythroblast-transformation-specific) transcription factors, resulting in 2–4-fold increases in TERT mRNA expression and telomerase activity [6,7,8]. This heightened telomerase activity correlates strongly with cancer cell proliferation rates, telomere length maintenance, and population doubling capacity [12,13,14,15]. Importantly, telomerase activity levels in melanocytic lesions demonstrate progressive elevation from benign nevi to primary melanomas and metastatic disease [4], suggesting a direct relationship with disease progression. Furthermore, telomerase activation has been implicated in therapeutic resistance to BRAF and MEK inhibitors [16], contributing to treatment failure and poor clinical outcomes [17].

Despite the well-established prevalence of telomerase activation in melanoma, several critical knowledge gaps persist regarding the relationship between telomerase activity levels and cancer cell population doubling time in melanoma [18,19]. Additionally, although telomerase inhibitors have demonstrated preclinical efficacy, their clinical translation has been limited by delayed cytotoxic effects and therapeutic resistance mechanisms that remain poorly understood [18,19,20]. Currently, no scoping review has systematically mapped the evidence linking telomerase activity to melanoma cell proliferation kinetics and doubling time. More literature about immunity and telomerase will be included in Section 3.

While this review focuses on melanoma, TERT promoter mutations represent a hallmark of telomerase activation across multiple human malignances. Similar mutations occur in approximately 70–80% of melanomas, 40–70% of glioblastomas, 60% of bladder cancers, and 50% of squamous cell carcinomas, suggesting conserved mechanisms of telomerase reactivation in cancer progression [20]. Understanding telomerase biology in melanoma thus provides insights that are applicable to other UV-exposed and non-UV-exposed cancers.

This scoping review aims to identify key studies by comprehensively synthesizing current knowledge and highlighting existing gaps. In doing so, the review will provide a foundation for future research directions and inform the development of telomerase-based strategies. The research team is notable for its international representation and includes experts in oncology, basic science, nursing, and pharmacy. This work will serve as a valuable reference for clinicians and researchers in the future. Drawing from the example of arsenic, when it was first submitted for use in medical treatment, few believed that such an apparently irrelevant emerging drug would one day become the cornerstone of leukemia treatment. Time will tell whether telomerase can become a useful target for refractory cases of melanoma once current well-known treatments have been exhausted.

## 2. Materials and Methods

A comprehensive search was conducted across four major electronic databases: Scopus, ScienceDirect, MEDLINE/PubMed, and CINHAL (Cumulated Index to Nursing and Allied Health Literature). It utilized a combination of keywords, including “telomerase”, “melanoma”, “cancer”, “cell proliferation”, and “doubling time”. We used the PRISMA-ScR (Preferred Reporting Items for Systematic reviews and Meta-Analyses extension for Scoping Reviews) methodology (registration number INPLASY2025120081).

### 2.1. Inclusion Criteria and Exclusion Criteria

The inclusion criteria were as follows: (1) quantitative, qualitative, or mixed-methods primary research on telomerase activity in melanoma and its role in cancer cell proliferation and doubling time, published in English between primarily January 2020 and July 2025, with select key studies from 2015 to 2019 included when providing essential mechanistic insights; (2) systematic and narrative review articles on telomerase in melanoma, published in English (any published date). While most included studies were published between January 2020 and July 2025, select key studies from earlier years (2015–2019) were retained because they elucidate important mechanisms of telomerase biology, drug resistance pathways, and therapeutic approaches that remain clinically relevant and are not superseded by more recent literature. This scoping review was conducted on studies published between January 2020 and July 2025. The landscape of telomerase-targeted therapies in the treatment of melanoma has evolved dramatically since 2020; therefore, we selected studies from the year 2020 onwards.

The decision to focus primarily on studies from 2020 to 2025 was based on the following scientific rationale:

1. Rapid therapeutic advances: The landscape of telomerase-targeted therapies in melanoma has evolved dramatically since 2020, with the emergence of novel agents (e.g., 6-thio-dG), CRISPR/Cas9 editing approaches, and next-generation immunotherapies. Our aim was to capture the most current evidence relevant to contemporary clinical practice.
2. Recent molecular discoveries: Key mechanistic insights, including the EXTEND algorithm, pan-cancer telomere maintenance mechanism (TMM) phenotypes, and TPP1 promoter mutations, were published within this timeframe and represent substantive advances over earlier work.
3. Selective inclusion of foundational studies: We explicitly included select studies from 2015 to 2019 (as stated in our inclusion criteria in Section 2.1) when they provided essential mechanistic insights that were not superseded by more recent literature. For example, foundational work on TERT promoter mutations and drug resistance pathways remains clinically relevant and continues to be cited in current research.
4. Scoping review purpose: Unlike exhaustive systematic reviews that aim for comprehensive historical coverage, scoping reviews map the current state of knowledge and identify gaps to guide future research. Our temporal focus aligns with this objective. Therefore, regarding the relatively low number of studies (*n* = 16), this reflects the novelty of specific therapeutic targets and the scarcity of direct doubling time data in this specific context, which is a key finding in itself that highlights the research gap we aim to explore.

Review articles were included because this is a scoping review with a methodological objective that is different from that of a systematic review. According to the PRISMA-ScR (Preferred Reporting Items for Systematic reviews and Meta-Analyses extension for Scoping Reviews) guidelines, scoping reviews aim to map the breadth and nature of existing evidence across heterogeneous study designs, including both primary research and secondary literature. In this context, we included select narrative and systematic reviews (*n* = 6 of 16 studies) for the following rationale:

1. Mapping conceptual frameworks: Reviews by Ali et al. and Tao et al. synthesized emerging therapeutic frameworks, e.g., the TICCA (Transient, Immediate, Complete and Combinatory Attack) strategy, that have not yet been validated in primary studies but represent important conceptual advances in the field.
2. Tracing primary evidence: Where reviews cited critical primary data, we systematically traced and reviewed the original articles to ensure accuracy. For example, mutation frequency data and mechanistic pathways were verified against primary sources.
3. Scoping review methodology: The Joanna Briggs Institute guidelines for scoping reviews explicitly state that including reviews alongside primary studies is appropriate when the goal is to comprehensively map available knowledge rather than synthesize effect sizes.

The exclusion criteria were as follows: studies unrelated to telomerase, those published in a language other than English, and review articles published before the inclusion window that do not provide critical mechanistic or clinical synthesis.

Two authors (O.A. and P.T.) independently reviewed study eligibility through title and abstract screening, followed by full-text assessment. Any discrepancies or disagreements were resolved through discussion and re-examination of the articles. A third researcher (A.D.) was available to arbitrate if a consensus could not be reached.

### 2.2. Quality Assessment and Data Analysis (Figure 1 and Tables S1–S3)

While a formal quality assessment such as the Mixed-Methods Appraisal Tool (MMAT) or Risk of Bias In Systematic Reviews (ROBIS) is typically reserved for systematic reviews, this scoping review evaluated sources’ credibility based on study design rigor and peer-review status. Data were synthesized using a thematic analysis approach, grouping findings into three core categories: (1) TERT promoter mutations and proliferation markers, (2) therapeutic targeting of telomerase, and (3) clinical prognostic correlations. These approaches allowed for identifying critical knowledge gaps regarding specific cell proliferation doubling times.

**Figure 1:**
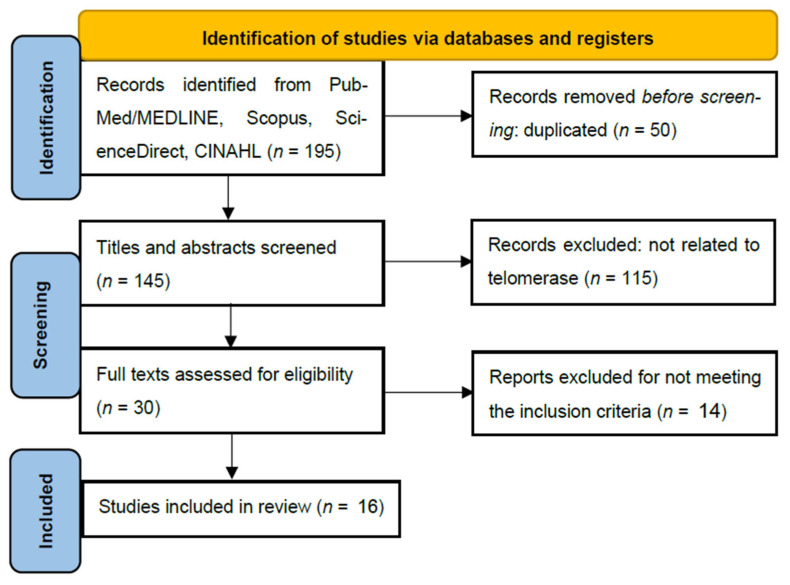
Flow diagram for PRISMA-ScR (PRISMA-ScR (Preferred Reporting Items for Systematic reviews and Meta-Analyses extension for Scoping Reviews)).

## 3. Results

Here, we summarize the common TERT promoter and gene alterations in melanoma, their effects on TERT expression and telomerase activity, associated tumor phenotypes, and potential implications for anti-TERT therapeutic strategies (Table 1).

### 3.1. TERT Promoter Mutations as Drivers of Proliferation

From Table 1, it can be seen that TERTp (TERT promoter) is important for diagnosis and prognosis. It may serve as a therapeutic target through telomerase inhibition or TERT-directed immunotherapies [28]. The findings support a two-step model of telomere-driven carcinogenesis. TERT promoter mutations (C228T, C250T) represent the initial ‘hit’ that increases telomerase activity. Critically, TPP1 (telomerase processivity protein 1), a core component of the shelterin complex that facilitates telomerase function and progressive telomere elongation, also exhibits promoter mutations in melanoma. Recent work demonstrated that dual UV-driven mutations in both the TERT and TPP1 promoters cooperatively remove ‘telomeric guardrails,’ acting synergistically to sustain telomere maintenance and drive melanoma progression. Sanford et al. conclude that dual promoter mutations in TERT and TPP1 remove the “telomeric guardrails” driving immortalization and tumor growth, while offering novel targets for telomerase- or ETS-based therapies [25].

Mechanistically, the most prevalent TERT promoter mutations (C228T and C250T, located 124 AND 146 bp upstream of the ATG start codon) create de novo binding sites for transcription factors of the ETS family, most notably GABP (GA-binding protein). GABP recruitment to these mutant promoters increases TERT mRNA expression 2–4-fold, resulting in elevated telomerase enzymatic activity [32]. This mechanism directly links TERT mutation status to functional telomerase levels and subsequent telomere maintenance and cancer cell proliferation.

From Table 2, it can be seen that TERT alterations in melanoma are associated with different phenotypes. Most TERT alterations are seen in melanoma cases, which result in increased TERT expression and elevated telomerase activity, particularly the highly recurrent promoter mutations C228T and C250T. These alterations cause de novo ETS transcription factor binding motifs, leading to transcriptional upregulation and promotion of proliferative immortality. Epigenetic activation through promoter hypermethylation in the TERT Hypermethylated Oncological Region (THOR), as well as gene amplification and structural rearrangements, provides additional mechanisms of telomerase induction. Collectively, the therapeutic implications of these changes support the biological rationale for anti-TERT therapeutic approaches, although predictive biomarkers of response remain to be fully validated.

Chun-On et al. identified somatic promoter variants in the TPP1 gene (shelterin complex) [31], which are present in 5–6% of cutaneous melanomas and frequently co-occurr with TERT promoter mutations [31]. These variants alter ETS transcription factor binding sites, increasing TPP1 expression. TPP1 promoter mutations thus represent a critical “second hit” cooperating with TERT activation to sustain telomere maintenance and drive melanoma progression. The findings highlight the importance of non-coding regulatory mutations and suggest new therapeutic targets [31].

**Figure 2:**
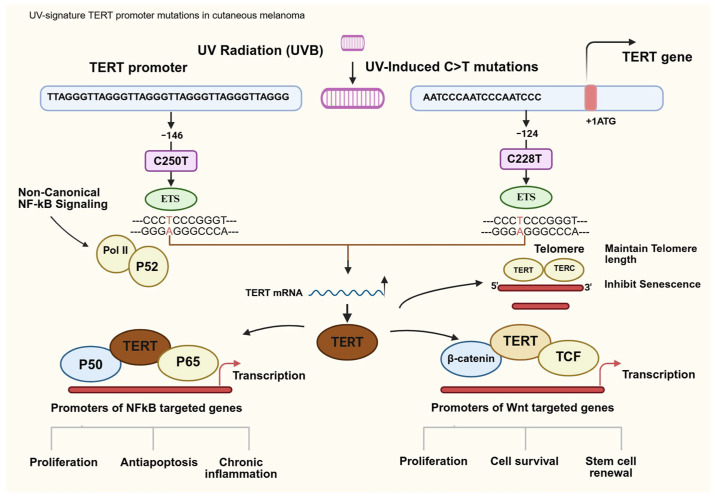
Telomerase reverse transcriptase promoter (TERTp) mutations in melanoma. Red fonts are mutated nucleosides.

In summary, across multiple studies, TERT promoter mutations were found to be a hallmark of cutaneous melanoma, representing high-frequency (70–80%) noncoding driver mutations, functional upregulation of telomerase, cooperation with MAPK signaling, and outcomes such as telomere stabilization, senescence bypass, and tumor persistence [7,8,16] (Figure 2, Tables S1–S3). These alterations act as a molecular signature of UV-induced carcinogenesis, offering diagnostic and prognostic value in tumor biology and therapeutic resistance. Table S1 summarizes the molecular mechanisms and mutation frequencies. The two canonical hotspot mutations in the TERT promoter are −124 C>T (C228T) and −146 C>T (C250T). Table S2 summarizes telomerase activity and proliferation studies. Because of these proliferative roles, telomerase and its regulation represent potential therapeutic vulnerabilities: targeting telomerase may reduce the proliferation of tumor cells. Table S3 summarizes the diagnostic value, targeted therapies, and clinical outcome of telomerase. Clinical trials have shown partial success: imetelstat has shown improvement and telomere shortening in some malignancies, but with hematologic toxicity concerns.

### 3.2. Diagnostic and Prognostic Significant of Telomerase in Melanoma

Telomerase is essential for telomere maintenance in stem cells and most cancers, but measuring its activity has been difficult [33]. EXTEND (EXpression-based Telomerase ENzymatic activity Detection) is a computational method that estimates activity from a 13-gene signature including TERT and TERC expression [15]. Validated across cancer cell lines, patient tumors, and non-neoplastic tissues, EXTEND outperformed TERT expression alone in predicting telomerase activity [15]. Applied to over 9000 tumors, it showed that telomerase activity varies by cancer type and correlates with stage, molecular subtype, oncogenic pathways, and prognosis [15].

Telomeres protect chromosome ends and regulate cell lifespan; however, in cancer, telomere maintenance mechanisms (TMMs) become dysregulated, enabling unlimited division [33].

### 3.3. Telomere Maintenance Mechanisms and Pathway Analysis

Two main pathways exist: telomerase-dependent (TEL) and alternative lengthening of telomeres (ALT) [34]. Hakobyan et al. analyzed RNA sequencing from 33 TCGA cancer types, categorizing tumors into five TMM phenotypes [24]. Normal tissues showed low activity, while cancers had higher TEL and ALT pathways, with ALT varying more widely [24]. Clinically, MSI-H tumors exhibited higher TMM activity, particularly in TEL and ALT branches. Survival analyses linked ALT-high/TEL-high and ALT-high/TEL-low phenotypes to poorer outcomes, suggesting that TMM activity drives tumor aggressiveness [24].

Several studies have delineated the diagnostic and prognostic value of TERT promoter mutations in advanced melanoma. Blanco-Gracia [35] conducted a retrospective analysis of 53 patients with advanced melanoma (88 tissue samples, 25 plasma samples) and identified striking prognostic differences based on TERT promoter mutation type:

- C250T variant (associated with 5.7-fold-increased TERT expression):
  - Progression-free survival (PFS): 5 months.
  - Overall survival (OS): 36 months.
  - Poorest prognosis among all groups.
- C228T variant (associated with 2.1-fold-increased TERT expression):
  - PFS: 23 months.
  - OS: 106 months.
  - Intermediate prognosis.
- Wide-type (no TERT promoter mutation):
  - PFS: 55 months.
  - OS: 223 months.
  - Best prognosis.

Despite the strong prognostic associations documented above, some heterogeneity exists in the literature regarding telomerase’s universal prognostic utility. In a comprehensive pan-cancer analysis of 11,123 samples from 33 cancer types, high telomere maintenance mechanism (TMM) activity—particularly ALT+/TEL+ and ALT+/TEL—phenotypes correlated with significantly worse overall survival [17]. Conversely, Bhari et al. argue that TERT expression or telomerase activity alone do not consistently predict clinical outcomes, noting substantial heterogeneity in prognostic associations across different tumor types and patient populations [36].

This apparent discrepancy likely reflects several factors: (1) methodology differences in telomerase measurement, (2) variations in patient populations, (3) cancer-type context, and (4) disease-stage-specific effects. In melanoma, variant-specific analysis (C250T vs. C228T vs. wild-type) appears to improve prognostic discrimination [35], suggesting that granular biomarker approaches may be more informative than the global ‘telomerase activity’ metric.

### 3.4. Therapeutic Targeting of Telomerase in Melanoma

Zhang et al. investigated 6-thio-2′-deoxyguanosine (6-thio-dG), a telomerase-directed nucleoside, as a treatment for therapy-resistant melanoma [26]. In pre-clinical models, 6-thio-dG selectively impaired telomerase-positive cancer cells while sparing normal skin cells [26]. In BRAF-mutated melanoma lines, it showed strong anti-proliferative effects, inducing telomere dysfunction, apoptosis, and senescence. In vivo, 6-thio-dG reduced tumor growth in xenografts without toxicity. Molecular analyses revealed suppression of telomere maintenance, cell cycle, DNA damage response and resistance markers such as AXL (AXL Receptor Tyrosine Kinase). Its efficacy depended on telomerase activity, as TERT depletion reduced sensitivity [26]. Tumor biopsies showed enrichment of telomere pathways in tumors progressing on targeted or immune therapies, implicating telomere reactivation in resistance [26]. The telomerase-directed agent 6-thio-dG impaired the growth of resistant melanoma cells and down-regulated proteins such as AXL, BRD4 (Bromodomain Containing 4), and ATM (Ataxia-Telangiectasia Mutated), highlighting its potential to overcome therapy resistance [26]. In BRAF-mutated melanoma, most patients carried TERT promoter mutations, with shorter progression-free survival than wild-type cases [16]. Resistant cell lines exhibited high TERT expression, driving MAPK reactivation independent of telomere lengthening [16]. Inhibiting TERT with 6-thio-dG reduced proliferation and, when combined with vemurafenib, suppressed resistant growth by up to 90%, in 3D models [16]. Therefore, TERT serves as both a biomarker and therapeutic target, and combining MAPK inhibitors with TERT-directed agents may improve outcomes. Telomerase inhibition remains attractive given its cancer specificity and minimal effects on normal cells [18].

Telomerase-targeted approaches include antisense oligonucleotides (e.g., imetelstat) that block telomerase RNA, inducing telomere shortening and apoptosis, and small-molecule inhibitors such as BIBR1532 or G-quadruplex stabilizers (telomestatin, BRACO-19, RHPS4) that disrupt telomerase binding [37]. Immunotherapies (hTERT vaccines, adoptive T-cell therapies) and gene therapy using telomerase promoters to drive cytotoxic proteins or oncolytic viruses (OBP-301) show promise, though immune tolerance and variability remain as challenges [38]. Recent studies on immunity in melanoma have identified a relationship between immunity and telomerase [39]. This will lead to more telomerase-based cancer vaccine development. One such trial is combination therapy with ipilimumab for the treatment of patients with metastatic melanoma [17].

Additional strategies include alternative splicing modulation to shift hTERT toward inactive isoforms, natural products (curcumin, resveratrol, EGCG) and off-target drugs (aspirin, rapamycin) with telomerase-inhibiting activity, and shelterin complex targeting (TRF1/TRF2 disruption) to induce telomere dysfunction [40]. CRISPR/Cas9 can edit hTERT or engineer immune cells, while ALT-positive tumors may respond to ATR inhibitors, G4 ligands, or ALT-specific oncolytic viruses [22].

Combination therapies enhance efficacy by pairing telomerase inhibition with chemotherapy, CRISPR, or dual-target molecules, while personalized therapy tailors treatment using biomarkers such as telomere length, ALT status, and tumor mutations [18]. The TICCA framework (Transient, Immediate, Complete, Combinatory Attack) integrates short-term inhibition, rapid telomere disruption, multi-pronged strategies, and combination regimens to overcome resistance [18].

Since 2010, multiple agents have entered clinical trials, including hTERT vaccines (GV1001, UV1, GX301, INVAC-1), telomerase inhibitors (imetelstat, KML-001), oncolytic viruses (OBP-301, KH901), and nucleoside analogues (6-thio-dG) [40]. Notably, 6-thio-dG induces telomere dysfunction, suppresses resistance markers, and enhances checkpoint blockade efficacy, while imetelstat has improved survival in NSCLC patients, supporting telomerase inhibition as a viable cancer therapy [29].

Clinically, C250T mutation is linked to poor prognosis, with shorter progression-free survival (5 months) and overall survival (36 months) compared to C228T (23 and 106 months) and wild-type tumors (55 and 223 months). High-TERT mRNA also predicted worse outcomes, but C250T remained the strongest biomarker [17,24]. Telomerase, particularly hTERT, contributes to chemo-resistance by maintaining telomeres and enhancing DNA repair [17,24]. Vault complexes and ABC transporters further support drug resistance, often regulated alongside hTERT by STAT5. Cancer stem cells (CSCs), with high telomerase and ABC transporter activity, are especially resistant, but telomerase inhibition (e.g., Imetelstat, BIBR1532, G-quadruplex stabilizers) can sensitize tumors to therapy [20]. Imetelstat is a first-in-class telomerase inhibitor, a lipid-conjugated oligonucleotide that targets the RNA template of telomerase (hTERC), thereby blocking telomere elongation.

In summary, telomerase activity drives resistance via telomere maintenance, mitochondrial protection, cancer stem cell survival, and transporter modulation, making it a critical therapeutic target [20].

### 3.5. TERT Genomic Alteration in Melanoma Culled from cBioportal-Based Studies

Multiple large melanoma groups analyzed through the cBioPortal platform have reported high frequencies of TERT alterations. The most common alterations include TERT promoter mutations (C228T and C250T), which result in high telomerase transcription. Additionally, copy number gains and amplifications of TERT have been documented, together with less frequent deep deletions and structural variants. These alterations collectively contribute to increased telomerase activity, increased proliferative capacity, and melanoma progression. Reported alteration frequencies across TCGA and other melanoma groups range approximately from 60% to 80% for promoter mutations, while copy-number gains/amplifications occur in a smaller but clinically relevant subset of tumors. These findings explain the strong selective advantage of telomerase activation in melanoma biology

## 4. Discussion

Telomerase has become central in cancer research, linking tumor biology with therapeutic innovation. Frequent TERT promoter mutations in melanoma, glioblastoma, and other cancers are among the most common noncoding mutations, providing a genetic mechanism for telomerase reactivation [6]. Once viewed only as a telomere-maintaining enzyme, telomerase is now recognized as a driver of immortality and regulator of pathways such as Wnt/β-catenin and NF-κB [9,40]. It is increasingly considered a prognostic marker, though predictive value varies by tumor type [29]. While Hakobyan et al. linked high activity to poor outcomes, others argue that telomerase does not always predict prognosis [24,35].

In melanoma, TERT promoter mutations occur in 70–80% of cases, strongly increasing telomerase activity and stabilizing short telomeres [6,7]. The model includes GABPα/β recruitment to ETS motifs and MAPK amplification, especially in BRAF V600E melanoma [32,41]. Yet, Heidenreich et al. noted that promoter mutations alone do not guarantee activity, as transcription factor dynamics, structural variants, and chromatin content also play roles [42]. In bladder cancer, TERT activation can occur via THOR hypermethylation without mutations [43], while breast and colorectal cancers often rely on gene amplification [35].

Understanding these pathways is vital for targeted therapies, as telomerase is a near-universal hallmark of advanced malignancy [44,45,46]. Resistance to BRAF and MEK inhibitors in melanoma is increasingly linked to TERT overexpression and promoter mutations, with patients showing shorter progression-free survival compared to wild-type variants [16]. Preclinical studies demonstrate that 6-thio-dG reduces growth in resistant models [16,26], supporting combined telomerase-targeted and MAPK inhibitor therapy. Future research should examine mutation-specific effects; Blanco García et al. identified that C250T variants predict worse survival than C228T or wild-type variants, underscoring the need for variant-level biomarker analysis in guiding treatment strategies [17].

This scoping review highlights a critical paradox in melanoma research: while telomerase activation via TERT promoter mutations is a well-established driver of immortality, precise quantitative data linking enzymatic activity levels directly to population doubling time remain scarce. Most studies rely on surrogate markers of proliferations, such as ki-67 index or tumor volume growth, rather than calculating specific doubling times.

The scarcity of direct doubling time correlations suggests that telomerase activity may function as a “permissive” factor rather than a direct accelerator of cell-cycle speed. As noted by Hakobyan et al., the co-activation of ALT and telomerase pathways complicates this relationship [24]. Tumors may maintain high proliferative potential without necessarily exhibiting a linear reduction in doubling time, possibly due to metabolic constraints or microenvironmental factors.

Studies utilizing cBioPortal datasets showed that TERT promoter mutations, copy-number alterations, and structural variants represent major mechanisms of telomerase reactivation in melanoma. Their presence corresponds to worse prognosis, increased tumor thickness, ulceration, metastasis, and poorer survival. More importantly, these genomic alterations may activate the response to immunotherapy and targeted therapy, suggesting clinical utility as prognostic and predictive biomarkers. Future research will integrate cBioPortal-driven genomic profiling with functional assays and clinical outcomes to clarify how specific categories of TERT alteration (promoter mutation vs. amplification vs. structural variant) differentially shape melanoma evolution and therapeutic vulnerability.

The current evidence does not consistently show major sex-based differences in TERT expression or promoter mutation frequency in melanoma. Apparent ethnic variations largely reflect melanoma subtype distribution, with TERT promoter mutations being common in UV-associated cutaneous melanoma but less frequent in acral and mucosal melanoma, which occur more frequently in darker-skinned populations. However, available data are limited by few representations of non-European groups, and further research is required to determine whether true biological differences exist.

Epigenetic alterations of TERT are present in melanoma. Promoter hypermethylation, particularly within the THOR, along with histone modifications adds to increased TERT expression. These epigenetic changes may occur independently of TERT promoter mutations or coexist with them, showing that genetic and epigenetic mechanisms can be combined to stimulate telomerase in melanoma.

The findings regarding 6-thio-dG and TERT-directed immunotherapy underscore the potential of targeting this pathway. However, the resistance mechanisms described by Delyon et al., particularly on MAPK pathway reactivation, suggest that telomerase inhibitors (e.g., imetelstat) will likely require coupling with BRAF/MEK inhibitors to be clinically effective [16]. The “TICCA” framework proposed by Ali and Walter represents a logical evolution of this strategy, moving from monotherapy to combinatorial attacks [18].

### 4.1. Limitations of This Study

One limitation of this review is its restriction to English-language publications, which may have introduced language bias by excluding relevant studies published in other languages. The review included only studies published between January 2020 and July 2025, potentially excluding relevant foundational work that could provide valuable historical context on telomerase biology in melanoma. The heterogeneity of the included study designs, ranging from narrative reviews to preclinical experimental studies and retrospective cohort analyses, limited formal quality assessment using standardized instruments such as MMAT or ROBIS, making direct comparisons challenging. While the included studies demonstrated the role of telomerase activity in telomere-dependent proliferation and therapy resistance, precise quantitively correlations between telomerase enzymatic activity levels and measured cell populations doubling times were sparse and inconsistently reported. Clinical outcomes data and survival analyses were limited, restricting conclusions about direct therapeutic relevance.

The review did not systematically examine tissue-specific variations across melanoma subtypes (cutaneous, acral, mucosal), which may influence applicability to them.

### 4.2. Future Research

* Prospective clinical trials directly measuring telomerase enzymatic activity alongside quantified cell population doubling times are essential to establish correlations between telomerase expression, telomere dynamics, and proliferation kinetics in vivo.
* Research should examine the mutation-specific effects of TERT promoter variants (C250T versus C228T) in influencing telomerase activity and cellular phenotypes, as variant level biomarkers may better predict therapeutic response.
* Investigators should characterize the extratelomeric functions of telomerase, including its role in mitochondrial metabolism, NF-kB signaling, and drug efflux transportation to identify synergistic opportunities for combined inhibition strategies.
* Comparative efficacy studies should evaluate emerging telomerase-directed strategies, including 6-thio-dG, imetelstat, G-quadruplex stabilizers, and the TICCA framework, within controlled clinical settings
* Future research should clarify the interplay between telomerase-dependent and ALT pathways in determining progression and resistance.
* Research must explore biomarker-driven approaches integrating telomere length, TERT mutation analysis, and immune checkpoint expression to guide personalized intervention strategies.

## 5. Conclusions

Telomerase is increasingly recognized as a central driver of melanoma progression through both telomere-dependent and -independent mechanisms. By maintaining telomere length, it enables unlimited proliferation and genomic stability, while its non-canonical roles influence signaling pathways, metabolism, and resistance to apoptosis. Frequent TERT promoter mutations, particularly C250T, highlight its prognostic significance and link telomerase activity to poor patient outcomes. Moreover, telomerase overexpression contributes to resistance against BRAF and MEK inhibitors, underscoring its role in treatment failure. Emerging therapies such as 6-thio-dG demonstrate the potential to disrupt telomere function and overcome resistance, especially when combined with MAPK inhibitors. The review identifies a significant gap in the literature regarding direct quantification of doubling times. Future studies should prioritize calculating specific kinetics parameters alongside enzymatic activity to move beyond static biomarkers towards a dynamic model of tumor growth.

## Figures and Tables

**Table 1:** The final 16 studies included in this scoping review to reflect on the current knowledge.

Author/ Reference	Purpose	Settings	Sample Size	Study Design	Main Findings
Boccardi [21]	To review the relationship between telomeres/telomerase, aging, inflammation and cancer.	Division of Gerontology & Geriatrics, U. Perugia (Italy) and affiliated medical/surgical depts, Poland.	N/A	Narrative review.	Describes how telomere shortening and telomerase dysregulation link chronic inflammation, aging and cancer, and summarizes telomerase-targeted therapies.
Guo [8]	To review the role of TERT promoter mutations and telomerase in melanoma biology, prognosis and treatment.	Dept of Oncology/Plastic & Burns Surgery, 1st Affiliated Hospital of Nanjing Medical U., China.	N/A	Narrative review.	Describes how hotspot TERT RT promoter mutations increase TERT/telomerase activity, link to aggressive disease and poor prognosis, Outlines emerging telomerase-targeted and combination therapies in melanoma.
Lipinska [20]	To review the mechanisms linking telomerase activity with drug resistance in cancer.	Dept of Clinical Chemistry and Molecular Diagnostics, Poznan U. of Medical Sciences, Poland.	N/A	Narrative review.	Describes how hTERT activation, mitochondrial translocation and vault/ABC transporter pathways contribute to chemoresistance and cancer cell survival.
Ali [18]	To summarize classical and novel approaches in telomerase-targeted cancer therapy and propose the TICCA concept.	Institute of Laboratory Medicine, Charité Berlin & University of Rostock, Germany.	N/A	Narrative review.	Reviews mechanisms of telomerase inhibition and introduces the TICCA strategy (Transient, Immediate, Complete, and Combinatory Attack) as a combined therapeutic model integrating CRISPR/Cas9, telomere deprotection, and hybrid inhibitors for improved long-term cancer control.
Tao [22]	To review the therapeutic potential of targeting telomere dynamics in cancer.	Institute of Medicinal Biotechnology, Chinese Academy of Medical Sciences, Beijing.	N/A	Narrative review.	Summarizes telomere- and telomerase-targeted drugs in clinical and preclinical stages, highlighting their chemotherapeutic and immunotherapeutic potential and integration into nanomedicine systems.
Welfer [23]	To summarize recent advances in human telomerase structural biology and implications for drug design.	U. Kansas Medical Center, Kansas City, USA.	N/A	Narrative review.	Reviews new cryo-EM structures of human telomerase, elucidating mechanisms of recruitment, telomere synthesis, and structural targets for rational inhibitor development.
Hakobyan [24]	To perform a pan-cancer analysis of telomere maintenance mechanisms (TMM) across 33 cancer types.	Institute of Molecular Biology NAS RA, Armenia; U. Leipzig, Germany.	11,123 TCGA samples.	Bioinformatics-based pan-cancer analysis.	Identified 5 distinct TMM phenotypes integrating telomerase (TEL) and ALT pathways; their coactivation correlated with worse survival and higher activity in MSI-H tumors.
Sanford [25]	To discuss how UV-induced promoter mutations in TERT and TPP1 cooperate to bypass telomere-based barriers in carcinogenesis.	U. Pittsburgh, USA.	N/A	Commentary/mechanistic review.	Highlights TERT and TPP1 promoter mutations as sequential UV-driven “hits” that cooperate to sustain telomere maintenance and melanoma progression.
Blanco-García [17]	To assess pTERT mutations/methylation in tissue and plasma of advanced melanoma and relate them to TERT expression and prognosis.	Hospital 12 de Octubre, Madrid, Spain.	53 pts (88 tumors; 25 plasma).	Retrospective cohort.	C250T mutation linked to higher TERT expression and poor survival; pTERT hypermethylation enriched in WT tumors.
Zhang [26]	To test telomerase-targeted nucleoside 6-thio-dG in therapy-resistant melanoma.	The Wistar Institute, Philadelphia, USA.	Several human melanoma cell lines and xenografts.	Preclinical experimental study.	6-thio-dG induces telomere dysfunction, apoptosis, and tumor control in therapy-resistant melanoma.
Robinson [27]	To review telomerase functions, regulation, and clinical applications in cancer.	Case Comprehensive Cancer Center, Case Western Reserve U., USA.	N/A	Comprehensive review.	Summarizes telomerase’s telomeric and extratelomeric roles, regulatory mechanisms, and its translational potential as a biomarker and therapeutic target.
Delyon [16]	To assess how TERT expression influences resistance to BRAF and MEK inhibitors in BRAF-mutated melanoma.	INSERM U976 and Hospital Saint Louis, U. Paris Cité, France.	48 patients + in vitro cell lines.	Translational and in vitro study.	High TERT expression correlated with reduced response to BRAF/MEK inhibitors; TERT overexpression reactivated MAPK pathway independently of telomere maintenance.
Sharma [28]	To summarize emerging molecular mechanisms underlying hTERT promoter–driven telomerase reactivation in cancer.	CSIR-Institute of Genomics and Integrative Biology, New Delhi, India.	N/A	Narrative review.	Describes how hTERT promoter mutations, chromatin looping, and G-quadruplex destabilization cooperatively reactivate telomerase across cancers.
Baylie [29]	To review telomere and telomerase structure, function, and their role as therapeutic targets in cancer.	Debre Markos U., Ethiopia.	N/A	Narrative review.	Summarizes telomerase biology and emerging therapeutic strategies, including antisense oligonucleotides, G-quadruplex stabilizers, and telomerase-targeted immunotherapies.
Kozyra [30]	To review newly synthesized anti-melanoma agents and their molecular targets (2020–2022).	Medical U. Lublin, Poland.	N/A	Systematic literature review.	Summarizes recent compounds targeting MAPK, PI3K–AKT, and ion-channel pathways; highlights benzimidazole-based telomerase inhibitors among emerging therapeutic candidates.
Chun-on [31]	To investigate whether TPP1 promoter mutations cooperate with TERT promoter mutations in melanoma.	U. Pittsburgh & UC Santa Cruz, USA.	749 melanoma samples.	Genomic and functional analysis.	TPP1 promoter variants co-occur with TERT mutations, enhancing TPP1 expression and synergistically lengthening telomeres in melanoma cells.
Noureen [15]	To quantify telomerase enzymatic activity and explore its link with cancer stemness and proliferation.	UT Health San Antonio & MD Anderson Cancer Center, USA.	>9000 tumors from TCGA and multiple validation cell lines.	Computational and experimental validation study.	Developed the EXTEND algorithm; showed telomerase activity strongly correlates with cancer stemness and proliferation, outperforming TERT expression as a biomarker.

ABC transporter pathway = ATP-binding cassette transporter pathway; ALT pathway = alternative lengthening of telomerase pathway; cryo-EM = cryo-electron microscopy; EXTEND algorithm = expression-based telomerase enzymatic activity detection; hTERT = human telomerase reverse transcriptase; N/A = not available; NAS RA = National Academy of Sciences Republic of Armenia; U = university; USA = United States of America; UT Health San Antonio = University of Texas Health San Antonio.

**Table 2:** Telomerase reverse transcriptase (TERT) alterations in melanoma, associated phenotype, and therapeutic implications. “THOR” stands for TERT Hypermethylated Oncological Region, a specific region within the TERT promoter that becomes hypermethylated in several cancers and is associated with telomerase activation.

TERT Alteration	Type of Alteration	Effect on TERT Expression/Activity	Cellular Phenotype Associated	Relationship with TERT Promoter Mutation	Potential Impact on Anti-TERT Therapies
C228T promoter mutation	Promoter mutation (ETS binding site creation)	↑ Increased TERT transcription and telomerase activity	Enhanced proliferation, replicative immortality, tumor progression	Independent or coexists with promoter methylation	Likely increased dependence on telomerase; potential better target for anti-TERT therapy
C250T promoter mutation	Promoter mutation (ETS binding site creation)	↑ Increased TERT transcription and telomerase activity	Increased tumor growth, invasion, poor prognosis	May coexist with THOR hypermethylation	May respond to telomerase-directed therapies (vaccines/inhibitors)
TERT promoter hypermethylation (THOR)	Epigenetic modification	↑ Upregulation of TERT despite hypermethylation	Telomerase reactivation and melanoma progression	Can occur with or without promoter mutations	Supports rationale for anti-TERT therapy; predictive value still under investigation
TERT gene amplification	Copy number gain	↑ Increased TERT expression due to higher gene dosage	Increased telomerase activity; aggressive biology	Independent of promoter mutations	Suggests strong telomerase dependence → potential sensitivity
Structural variants near TERT	Rearrangements	↑ Increased enhancer–promoter interactions	Telomerase activation and tumor progression	Sometimes independent of promoter mutation	Mechanism-specific response unknown; still investigational
Wild-type TERT promoter with low methylation	No mutation; no THOR methylation	→ Low or baseline activity	Reduced telomerase dependence	None	May show less benefit from anti-TERT-directed therapy

↑ = Increases; → = no consistent change.

## Data Availability

No new data were created or analyzed in this study. Data sharing is not applicable to this article.

## Supplementary Materials

The following supporting information can be downloaded at: https://www.mdpi.com/article/10.3390/curroncol33020074/s1, Table S1: Summary of molecular mechanism and mutation frequencies [8,25,28,31]; Table S2: Summary of telomerase activity and proliferation studies [15,21,24,27]; Table S3: Telomerase—Diagnostic value, targeted therapies and clinical outcome [16,17,18,20,26,29].

## Abbreviations

ABC	ATP-binding cassette
ALT	Alternative lengthening of telomeres
BRAF	v-Raf murine sarcoma viral oncogene homolog B
BRAF V600E	A common activating mutation in the BRAF gene that leads to constitutive activation of the MAPK pathway
CRISPR	Clustered regularly interspaced short palindromic repeats
EGFR	epidermal growth factors receptor
EXTEND	Expression based telomerase enzymatic activity detection
ETS	E26-transformation-specific (Erythroblast-transformation-specific)
G1-S/G2-M phases	Cell-cycle phases
GABP	GA-binding protein
GO	Gene ontology
hTERT	Human telomerase reverse transcriptase
MAPK	Mitogen-activated protein kinase
MEK	Mitogen-activated protein kinase
miRNA	Micro-ribonucleic acid
mRNA	Messenger ribonucleic acid
MSI-H	Microsatellite instability-high
N/A	Not available
NF-kB	Nuclear factor kappa-light-chain-enhancer of activated B cells
NF1	Neurofibromin
NRAS	Neuroblastoma RAS viral oncogene homolog
PMCA4	Plasma membrane calcium ATpase4
PSF	Pathway signal flow
RNA-seq	RNA sequencing
ROS	Reactive oxygen species
SEER	Surveillance, epidemiology and end results
TCGA	The cancer Genome atlas
TEL	Telomerase-dependent pathway (telomerase-positive phenotype)
TERT	Telomerase reverse transcriptase
TF	Transcription factor
TICCA	Transient, immediate, complete and combinatory attack
TME	Tumor microenvironment
TMM	Telomere maintenance mechanism
TPP1	Telomerase processivity protein 1
UV	Ultraviolet
VEGF	Vascular endothelial growth factor
WT	Wild type
