# Supplementary material for: Telomerase Activity in Melanoma: Impact on Cancer Cell Proliferation Kinetics, Tumor Progression, and Clinical Therapeutic Strategies—A Scoping Review
Source: Curr Oncol. 2026 Jan 27;33(2):74. doi: 10.3390/curroncol33020074 (PMC12939225; doi:10.3390/curroncol33020074)
Supplement: Supplementary file 1 [file curroncol-33-00074-s001.zip › curroncol-4055580-supplementary.pdf]

Table S1: Summary of molecular mechanism and mutation frequencies.

Study	TERT mutation types	Mutation frequency	Fold increase in activity	Key molecular pathway
Guo [8]	−124 C>T (also −124/125 CC>TT), −146 C>T, −138/139 CC>TT, −136 C>T, −100 C>T, germline −57 A>C among others.	Exceed the frequency of any known noncoding mutations in melanoma but gives no single % for all melanoma; hotspot earlier studies show up to ~70-80% in some cutaneous melanomas.	Reported ~1.5–4-fold increase in some early functional studies; review notes increased mRNA expression, telomerase activity and telomere length, but a precise consistent fold for all is not always given.	Creates novel ETS transcription factor binding sites → recruitment of ETS/GABP complexes → increased TERT transcription → elevated telomerase activity → telomere length maintenance → melanoma cell immortalization & progression.
Sharma [28]	−124 C>T (C228T) and −146 C>T (C250T) are the two canonical hotspot mutations in the *TERT* promoter.<br>Both create de novo ETS transcription factor binding sites (GGAA motifs), especially recruiting GABPA. Occasionally, rarer mutations (e.g., −57 A>C, small insertions) have been reported but are un-common in melanoma	Reported in ~70–80% of cutaneous melanomas (primary and metastatic).<br>More frequent in tumors with UV-signature mutations and BRAF V600E or NRAS driver mutations.<br>Much lower in acral/mucosal melanomas (~10–15%).	Promoter-reporter assays show a 2–4.5-fold increase in *TERT* transcription with −124C>T or −146C>T compared to wild-type promoter.<br>Endogenous *TERT* mRNA levels can be 10–20× higher in mutation-positive melanomas relative to normal melanocytes.<br>Telomerase enzymatic activity increases modestly (~2×) compared with wild-type lines	In melanomas with BRAF V600E, the MAPK (RAS–RAF–MEK–ERK) pathway further enhances *TERT* transcription by phosphorylating and activating ETS factors.
Sanford [25]	−124 C>T; −146 C>T (also −138/−139 CC>TT	~70–75% of cutaneous melanomas (highly recurrent).	Reporter and expression studies report ~2–4-fold increases in TERT promoter activity / expression for the common −124/−146 mutants (values vary by assay).	Mutations create de-novo ETS transcription factor binding motifs (e.g., recruit GABP/GABPA or ETS factors), upregulate TERT → increased telomerase activity → extension of shortest telomeres & partial bypass of replicative senescence (but alone may not prevent bulk telomere shortening).

Chun-On [31]	−124 C > T; −146 C > T (also −138/−139 CC > TT) creating de novo ETS family TF binding sites<br>−108 C > T; −75 C > T promoter variants (create/modify ETS sites)	70-75% of cutaneous melanomas (≈75%)	~2- to 4-fold increase in transcriptional reporter assays (for −124/−146)	Activation of ETS transcription factors (e.g., GABPA/GABPB) → increased TERT expression → increased telomerase activity → delayed telomere shortening / replicative senescence bypass.

ALT: Alternative Lengthening of Telomeres; ABC: ATP-Binding Cassette; BRAF: v-Raf Murine Sarcoma Viral Oncogene Homolog B; CRISPR: Clustered Regularly Interspaced Short Palindromic Repeats; ETS: E26 transformation specific (Erythroblast transformation specific); hTERT: Human Telomerase Reverse Transcriptase; MAPK: Mitogen-Activated Protein Kinase; MEK: Mitogen-Activated Protein Kinase; miRNA: Micro-Ribonucleic Acid; MSI-H: Microsatellite Instability-High; NF1: Neurofibromin 1; NRAS: Neuroblastoma RAS Viral Oncogene Homolog; PMCA4: Plasma Membrane Calcium ATPase 4; SEER: Surveillance, Epidemiology and End Results; TCGA: The Cancer Genome Atlas; TEL: Telomerase-Positive Phenotype; TERT: Telomerase Reverse Transcriptase; TICCA: Transient, Immediate, Complete and Combinatory Attack; TME: Tumor Microenvironment; TMM: Telomere Maintenance Mechanism; TPP1: Telomerase Processivity Protein 1; UV: Ultraviolet; WT: Wild Type, N/A: not available.

Table S2: Summary of telomerase activity and proliferation studies.

Study	Sample size	Method / analysis	Key findings on proliferation
Noureen [15]	The study analyzed over 9,000 tumor samples from The Cancer Genome Atlas (TCGA) and also included single-cell RNA sequencing data (glioblastoma, head & neck cancer, medulloblastoma) for validation.	Developed a gene-expression based signature method called EXTEND (EXpression-based Telomerase ENzymatic activity Detection), **c**omprising 13 genes, to estimate telomerase enzymatic activity across samples. Correlated the EXTEND score with measures of "cancer stemness" (derived from embryonic stem cell signatures) and proliferation markers (e.g., MKI67).	A strong positive correlation between estimated telomerase activity (via EXTEND) and proliferation: across cancer types the correlation with proliferation marker MKI67 was high (Rho ~0.9, $P = 2.5\times10^{-11}$) in many tumor cohorts. Small population of tumor cells with both high telomerase activity and stemness also were cycling cells (G1–S or G2–M phases) in single-cell analyses.
Hakobyan [24]	Analyzed RNA-sequencing data from The Cancer Genome Atlas (TCGA) covering 33 different cancer types.	Computed pathway signal flow (PSF) activity scores for two major telomere-maintenance mechanism (TMM) pathways: telomerase-dependent (TEL) and alternative lengthening of telomeres (ALT). Using the PSF scores tumors were stratified into 5 phenotypic groups (ALThigh^\mathrm{high}high/TELlow^\mathrm{low}low, ALTlow^\mathrm{low}low/TELlow^\mathrm{low}low, ALTmiddle^\mathrm{middle}middle/TELmiddle^\mathrm{middle}middle, ALThigh^\mathrm{high}high/TELhigh^\mathrm{high}high, ALTlow^\mathrm{low}low/TELhigh^\mathrm{high}high). Gene-ontology (GO) enrichment analyses were carried out for each phenotype to evaluate associated biological processes (including cell cycle regulation, DNA replication, chromosome dynamics) and evaluated associations with clinical parameters/outcomes	TEL- and ALT-associated phenotypes differ in their enrichment of proliferation and cell-cycle processes: e.g., GO terms in TEL-high phenotypes were strongly enriched for "DNA replication" and "cell cycle" functions, indicating that high telomerase pathway activity correlates with enhanced proliferative programs. Furthermore, the authors show that certain TMM phenotypes correspond to worse patient outcomes, supporting the link between telomere maintenance, proliferation and tumor aggressiveness.
Boccardi [21]	A narrative review, not a primary experimental study; therefore, it has no single sample size of participants or cell-lines reported. The authors described performing a literature search (in databases such as PubMed/Medline/Cochrane) to identify	Conducted a structured literature search using key-words ("aging", "cancer", "older", "telomere", "telomerase", "inflammation", "health") in PubMed, Medline and Cochrane, and included studies from bench to clinical trials. Findings were synthesized under thematic domains: telomere/telomerase biology; telomerase in aging; inflammation's impact on telomerase; and therapeutic strategies linking these. Analyses are qualitative/narrative rather than quantitative meta-analysis; the review	The review points out that TERT does more than just elongate telomeres: it has non-canonical roles that contribute to proliferation, such as enhancing mitochondrial function, reducing oxidative damage, interacting with signaling pathways (Wnt/β-catenin, NF-κB) and growth factor regulation (e.g., VEGF) which together boost cell growth and survival. In the context of aging and inflammation: chronic inflammation and oxidative stress can suppress telomerase in normal tissue, accelerate telomere shortening, promote senescence, and thus suppress proliferation in aging tissues. Conversely, in transformed cells, telomerase reactivation allows

	relevant publications up to ~7 July 2024.	outlines mechanistic pathways, historical and recent evidence, and points to translational opportunities (rather than pooling raw data). For example, they discuss mechanistic studies of how telomerase (specifically the catalytic subunit TERT) influences proliferation, mitochondrial function, ROS, inflammatory signaling (e.g., NF-κB) in aging/cancer contexts.	escape from senescence and enables sustained proliferation. The interplay implies that telomerase promotes proliferation in the cancer context through telomere maintenance + signaling augmentation; whereas in aging/inflamed tissue the lack of telomerase and telomere attrition limits proliferation (or forces senescence). The review suggests that therapies targeting telomerase may reduce proliferation in cancers, by impairing telomere maintenance and the non-telomeric proliferative signals, thereby limiting cell growth.
Robinson [27]	This is a review article, not an original experimental study, so there is no single sample size of patients or experiments reported. The review draws on numerous primary studies (cell lines, animal models, clinical tumor specimens) across many cancer types, but does not aggregate them into a meta-analysis with combined sample size data. Thus, for the purpose of studying proliferation, individual cited studies have their own sample sizes, but the review itself does not present a unified "n".	The narrative literature review was conducted, summarizing the current state of knowledge on telomerase (particularly the catalytic subunit TERT and the RNA component TERC) in cancer: its regulation (transcriptional, post-transcriptional, post-translational), telomere-dependent and telomere-independent (extratelomeric) functions, clinical relevance (biomarkers, therapeutic targets). They synthesize mechanistic findings (e.g., pathways controlling TERT expression, splice variants, non-canonical functions), refer to clinical/translational work (telomere length measurement, telomerase inhibitors, immunotherapies) and discuss gaps & future directions. No quantitative meta-analysis (statistical pooling) is performed; rather the authors integrate diverse mechanistic and translational studies qualitatively	One of the central threads is that activation of telomerase (TERT) in cancer cells underpins their ability to maintain telomeres, thereby supporting unlimited proliferative capacity. The review emphasizes that telomerase activation is ***a key*** enabler of proliferative immortality in cancer: malignant cells often reactivate telomerase to maintain telomere length, thereby avoiding replicative senescence and supporting continuous division. It highlights that beyond telomere elongation, telomerase (especially TERT) has extratelomeric functions that directly promote cancer cell proliferation. For example: TERT can act as a transcriptional co-factor: binding promoters of growth/survival genes (e.g., VEGF, EGFR), up-regulating signalling pathways (Wnt/β-catenin, NF-κB) which drive proliferation. TERT also modulates cellular metabolism/mitochondrial function (reducing ROS, supporting mitochondrial genome integrity), thereby enhancing cancer cell survival and growth capacity. Thus, proliferation is enhanced both by the canonical telomere maintenance role of telomerase (allowing further cell cycles) *and* by non-canonical roles that boost growth signalling and metabolic fitness. The review also suggests that because of these proliferative roles, telomerase and its regulation represent potential therapeutic vulnerabilities: targeting telomerase may reduce proliferation of tumor cells. However, the authors note that translation to clinic has been modest so far.

TERT :Telomerase Reverse Transcriptase, *ETS* : E26 Transformation *GABP* : GA-Binding Protein, *mRNA* : Messenger Ribonucleic Acid,,*MAPK* : Mitogen-Activated Protein Kinase , *BRAF V600E* : A common activating mutation in the *BRAF* gene that leads to constitutive activation of the MAPK pathway, promoting tumor growth and *TERT* transcriptional upregulation. *NRAS* : Neuroblastoma RAS Viral Oncogene Homolog *UV* : Ultraviolet, *TF*: Transcription Factor

Table S3: Telomerase—Diagnostic value, targeted therapies and clinical outcome.

Study	Therapeutic agent /strategy	Study type	Outcome
Zhang [26]	The study uses the telomerase-directed nucleoside 6-thio-2′-deoxyguanosine (6-thio-dG) to induce telomere dysfunction in therapy-resistant melanoma cells	Preclinical experimental study: in vitro and in vivo melanoma models (human and mouse), specifically focused on therapy-resistant melanoma cell lines and xenografts.	6-thio-dG induced telomere dysfunction, apoptosis and cell death in therapy-resistant melanoma models, and significantly prolonged disease control in vivo in resistant melanoma models. It also down-regulated AXL expression (a resistance associated receptor) in treated cells.
Delyon [16]	Overexpression of TERT to model resistance; (2) Use of BRAF inhibitor (vemurafenib) and MEK inhibitor (cobimetinib) in BRAF-mutated melanoma cells; (3) Inhibition of telomerase via a telomerase-dependent substrate (6-thio-dG) to assess reversal of resistance.	Translational + in vitro experimental study: A cohort of 48 advanced BRAF-mutated melanoma patients was analyzed for TERT promoter mutation status and TERT expression. In vitro cell-line work: BRAF-mutated melanoma cell lines (A375, SkMel28) & derived resistant lines; overexpression of TERT; assays for proliferation, EC_{50} determination, ERK phosphorylation.	Higher TERT expression (and TERT promoter mutation) tended to associate with shorter PFS in BRAF/MEK inhibitor-treated melanomas. (median PFS wild-type promoter ~13 months vs mutated ~4–5.6 months). In vitro, TERT overexpression caused **31- to 80-fold** increased resistance to vemurafenib and **20- to 55-fold** increased resistance to cobimetinib compared to parental cells. Mechanistically, TERT overexpression maintained ERK phosphorylation under BRAF/MEK inhibition, indicating MAPK pathway reactivation independently of telomere-lengthening function. Inhibition of telomerase substrate 6-thio-dG reduced proliferation of both parental & resistant cell lines, and combination of 6-thio-dG with BRAF inhibitor achieved ~90% reduction in proliferation in resistant models
Ali [18]	***TICCA strategy***: a proposed framework for telomerase-based therapy comprising “Transient, Immediate, Complete and Combinatory Attack” i.e. combining telomerase inhibition (or telomere-deprotection) with other concurrent modalities, timing the intervention (transient vs long-term), ensuring complete telomere attrition or damage, and using combinatory approaches (e.g., telomerase + ALT targeting + conventional therapies).	Review article (conceptual/theoretical)	Monotherapy targeting telomerase so far had limited clinical translation because of the slow kinetics of telomere shortening, compensatory alternative lengthening of telomeres (ALT) mechanisms and limited specificity. They propose that adopting a TICCA approach could improve long-term survival by: (1) transiently inhibiting telomerase to avoid selection/resistance, (2) immediate damage to telomeres or deprotection, (3) complete telomere collapse rather than gradual attrition, and (4) combination with other therapies to both shorten and damage telomeres

			and kill cancer cells. The review thus outlines the conceptual outcome: repositioning telomerase-based therapy into a multi-modal, timed treatment rather than a stand-alone long-term inhibitor
Blanco-Garcia [17]	None in the sense of an active therapy. This is a biomarker/ prognostic study. It examines the promoter mutation of TERT (specifically the C250T variant) in metastatic melanoma, and assesses its association with outcome.	A retrospective observational molecular-clinical correlation study analyzed tumor tissue (FFPE samples) and plasma cfDNA of stage III/IV) melanoma. Total tumor samples: 88 from 53 patients; plasma: 25 patients.	The TERT promoter C250T mutation was significantly associated with worse PFS and overall survival (OS) compared to the C228T mutation or wild type. Specifically: C250T had HR ~5.85 for OS vs wild type (p=0.001) and HR ~5.11 for PFS (p=0.004). Also, C250T tumors showed higher TERT mRNA expression compared to C228T
Lipinska [20]	The central therapeutic strategy discussed is targeting telomerase (particularly its catalytic subunit, hTERT) in cancer — either by inhibiting its activity or by blocking its non-telomeric functions.<br>Key strategic elements include: use of telomerase inhibitors (e.g., BIBR1532, imetelstat) to sensitize cancer cells to chemotherapeutic agents. Targeting telomere maintenance mechanisms (telomerase, telomere length regulation, alternative lengthening of telomeres – ALT) to overcome drug resistance. Recognizing non-canonical roles of telomerase (mitochondrial localization of hTERT, involvement in anti-apoptotic, ROS/mitochondria regulation, ABC efflux pump expression) as contributing to resistance, and thus potential secondary targets.	A narrative review, synthesizes existing pre-clinical & experimental studies (in vitro cell lines, some in vivo) exploring the relationship between telomerase activity and drug resistance across multiple cancer types. It does not report its own primary clinical trial data, but rather aggregates mechanistic evidence and therapeutic implications.	High telomerase expression/activity is highly correlated to reduced sensitivity to certain chemotherapeutic agents (e.g., doxorubicin, cisplatin, 5-fluorouracil). Inhibiting telomerase (pharmacologically or via gene knockdown) can **sensitize** resistant cancer cells to therapy in some settings, e.g. inhibition of telomerase in breast cancer cells increased sensitivity to doxorubicin. Several mechanistic pathways are implicated in this resistance link: hTERT translocation to mitochondria modulates ROS levels and mitochondrial DNA content, reducing apoptosis. Telomerase activity influences genome stability, telomere length, influencing how cells respond to DNA-damaging therapies. Some cancer stem cell subpopulations show high telomerase, high ABC transporter expression, contributing to intrinsic drug resistance
Baylie [29]	(1) Telomerase inhibitors: Imetelstat (GRN163L), an oligonucleotide that binds the RNA template region of telomerase (hTR), blocking telomere elongation, leading to progressive telomere shortening and apoptosis.<br>(2) G-quadruplex stabilizer: small molecules such as telomestatin and	A comprehensive narrative review, not a clinical or experimental study, synthesizes existing preclinical and clinical evidence on telomere biology, telomerase function,	Telomerase activation is a hallmark of cancer that enables unlimited proliferation, making it a promising therapeutic target. Preclinical data demonstrate that telomerase inhibition or telomere destabilization effectively reduces tumor growth and induces senescence or apoptosis. Clinical trials

	BRACO-19 that stabilize telomeric G-quadruplex structures, preventing telomerase access to telomeres and disrupting replication. (3) Immunotherapies: Telomerase-based vaccines (GV1001, GX301, UV1) stimulate cytotoxic T lymphocyte responses against TERT-expressing cancer cells, being evaluated for melanoma, lung & pancreatic cancers. (4) Gene therapy/oncolytic strategies, hTERT promoter–driven oncolytic viruses selectively replicate in telomerase-positive cells, inducing tumor-specific lysis, e.g. OBP-301 (Telomelysin) exploits high telomerase activity in tumor cells. (5) Telomere dysfunction inducers: 6-thio-2′-deoxyguanosine (6-thio-dG) incorporates into telomeres, inducing dysfunction & DNA damage responses selectively.	and therapeutic interventions targeting these mechanisms in cancer. Sources include molecular studies, in vitro cell-based assays, in vivo animal models, and human clinical trials assessing telomerase-targeted therapies.	show partial success: Imetelstat has shown improvement and telomere shortening in some malignancies but with hematologic toxicity concerns. Telomerase vaccines (e.g., GV1001, GX301, UV1) demonstrated safety and immunogenicity, with modest improvements in survival in select cancers. Combination therapies—integrating telomerase inhibition with chemotherapy, radiotherapy, or immune checkpoint blockade may enhance treatment efficacy. Future directions include refining telomerase-targeting specificity, minimizing toxicity, and exploring telomerase's noncanonical roles in signaling and metabolism as potential intervention points

ALT: Alternative Lengthening of Telomeres; EGFR: Epidermal Growth Factor Receptor; EXTEND: EXpression-based Telomerase ENzymatic activity Detection; G1–S/G2–M phases: Cell Cycle Phases; GO: Gene Ontology; hTERT: human Telomerase Reverse Transcriptase, the catalytic protein subunit of telomerase in human cells; mRNA: Messenger Ribonucleic Acid; NF-κB: Nuclear Factor kappa-light-chain-enhancer of activated B cells; PFS: progression-free survival PSF: Pathway Signal Flow; RNA-seq: RNA Sequencing; ROS: Reactive Oxygen Species; TCGA: The Cancer Genome Atlas; TEL: Telomerase-dependent pathway; TERT: Telomerase Reverse Transcriptase; TICCA strategy: Transient, Immediate, Complete and Combinatory Attack; TMM: Telomere Maintenance Mechanism; VEGF: Vascular Endothelial Growth Factor.
